# Awaken Immune Cells by Hapten Enhanced Intratumoral Chemotherapy with Penicillin Prolong Pancreatic Cancer Survival

**DOI:** 10.7150/jca.82985

**Published:** 2023-05-08

**Authors:** Baofa Yu, Yan Han, Qiang Fu, Feng Gao, Peng Jing, Zheng Guoqin, Peicheng Zhang, Jianbo Huang, Jian Zhang

**Affiliations:** 1 Jinan Baofa Cancer hospital, Jinan, Shandong Province, China, 250000.; 2 Beijing Baofa Cancer Hospital, Beijing, China, 100010.; 3 Immune Oncology Systems, Inc, San Diego, CA, USA, 92102.; 4 Huanan Hospital, Shenzhen University, Shenzhen, Guangdong, China, 518055.; 5 TaiMei Baofa Cancer hospital, Dongping, Shandong Province, China, 271500.

**Keywords:** Penicillin, Intratumoral injection, Cancer immunotherapy, Drug delivery, Extracellular matrix as a drug carrier, Autologous coagulum, Intracellular drug delivery

## Abstract

Intratumoral immunotherapy is well studied and is ongoing, but few studies have evaluated the relationship between of cytotoxic drugs intratumoral injection (CDI) and hapten-enhanced cytotoxic drugs intratumoral injection (HECDI) and patient survival. The objectives of this study include comparisons to explore possible associations between the proportions of treatment-induced cytokines and autologous antibodies to tumor-associated antigens (TAAs) and the relative size of the abscopal effects concurring. CDIs contain oxidant and cytotoxic drugs, HECDIs contains the same drug plus penicillin as the new Hapten. Of the 33 patients with advanced pancreatic cancer, 9 received CDI, 20 received HECDI, and 4 (control group) received placebo. Serum levels of cytokines and autoantibodies of TAAs were detected and compared after therapy. The 1-year survival rate was 11.11% for CDI and 52.63% for HECDI (P= 0.035). In the general analysis of cytokines, HECDI exhibited an increasing level of IFN-γ and IL-4, and the non-hapten CDI showed a rising level of IL-12 (P = 0.125, 0.607, & 0.04). Participants who did not receive chemotherapy had significant differences in the level of Zeta autoantibody only before and after HECDI; However, IMP1 levels in patients with previous chemotherapy experience were significantly different before and after HECDI and CDI treatment (P≤0.05, P = 0.316). After HECDI treatment, TAA autoantibodies of RalA, Zeta, HCC1, p16 increased (P = 0.429, 0.416, 0.042, 0.112). The elevated levels of CXCL8, IFN-γ, HCC1, RalA, Zeta, and p16 observed in HECDI may be attributed to the abscopal effect (P = 0.012 & 0.013). Overall survival rates indicated that HECDI treatment extended participants' lives.

## Introduction

The abscopal effect is a hypothesis for treating non-irradiation tumor after local radiation therapy 1. The mechanism of this phenomenon is still unknown since 1953, Mole mentioned an overview in the phrase abstract more than half a century 1. The abscopal effect is rarely witnessed in clinical 2. The uptick alludes to humans' growing tolerance to tumor antigens and a subsequent decline in our immunological defense against metastasis 2. However, a promising new research was observed that abscopal is associated with products of tumor-associated gene expression as autoantibodies (aTAAs) in reaction to the tumor-associated antigens (TAAs), with increasing of anti-MAGEA3 after localized radiation therapy 4 and a relationship between the abscopal effect and an immune response, it associates with antibodies and cytokines of immunity systems in the body 5.

The use of radiation therapy is believed to trigger the effect; however, it rarely results in a broad-spectrum regression of tumors 6. Therefore, by focusing on clinical immune checkpoint inhibitors' ability to activate the abscopal effect, scientists are fleshing out more effective immune therapeutic approaches 7. Such inhibitors reduce T-cells' dysfunction, ultimately preventing premature immunological inactivity before the aggregate destruction of cancerous cells 7. Many similar studies related abscopal effect were carried out, for example, co-injection intratumoral injection of poly I: c derivative BO-112 and STING agonist acted synergistically to achieve local and distant antitumor effects 8; TLR9 agonists intratumoral injection promotes immune permitting microenvironmental transformation and in combination with anti-PD1, produces synergistic antitumor activity in pancreatic cancer 9; oligonucleotide STNM01 on tumor growth was studied intratumroal injection for patients with unresectable pancreatic cancer^10^; a different studies have been conducted in all aspects of immunotherapy and drug therapy, including the effects of injection techniques, drug formulations, and tumor microenvironment on intratumoral immunotherapy delivery and efficacy, oncolytic viruses, injectable gels, antihypertensive nanoblockers, and multifunctional platinum-drug delivery silicon nanocarriers for effective chemotherapy immunotherapy of pancreatic cancer 11-15. However these studies were limited to single drugs or immunosuppressants or agonists, our study attempts to take advantage of a patient's tumor killing to produce a multi-tumor antigen related effect of immunotherapy.

Clinically we seldom witness the abscopal effect 16, since most of cancer patients got the standard of care with concurrent chemo or radiotherapy (CCRT), the immune system was damaged to a low function of immune T cells or T cells in week to sick (WtoS), and lose capability to recognize tumor antigens, thus the immune response is weaken and unable to strength the T cell in WtoS condition for fighting tumor cells. Improving the situation of WtoS T cell is very important for the immune therapy, firstly it should avoid a higher dose of chemotherapy or radiation therapy for late stage of cancer patients in order to prevent the function of immune cells, and secondly, awakening the immune cells in fighting posture is very important too, so that we have tried to use hapten enhanced cytotoxic drugs intratumoral injection (HECDI) in order to waken the immune cell out of WtoS to recognize tumor cells and make a patient's immune system ready for immune therapy, also just cytotoxic drugs intratumoral injection (CDI) as control. Since HECDI can increase T-cells' likelihood of fighting tumor earlier, these drugs and immune responses are highly adept at suppressing tumor recurrence to prevent and reduce the metastasis 17, 18. HECDIs contain a clinically approved oxidant (H2O2) plus cytotoxic drugs (Cytarabine & Doxorubicin) and penicillin as hapten 19-21.

Hapten, a low molecular weight (<1000 Dalton) chemical that combines with carrier molecules to establish or enhance antigenicity, is proficient at targeting proteins like tumor associate antigens (TAAs) 22, 23. Haptenization, the combination of cytotoxic drugs and a hapten, produces potent tumor antigens more vulnerable to the immune system, increasing the likelihood that one's body will undergo an abscopal effect 22, 23.

Furthermore, due to the hapten inlay in the tumor with cytotoxic drug, enhanced systemic immunity is enabled, resulting in the manifestation of vaccine-like effects on tumors 24. When various autologous tumor antigens are released from tumor killed by drugs and haptenized multiple tumor antigens as polyvalent vaccine, the cell death may trigger T and B cells response and induce effective immunity 25. The cell deaths, or "good deaths," act as immunologic modulators (i.e., small molecule haptens embedded in denatured tumors) to bolster an in-vivo self-vaccination in the body 26. Previously published clinical and animal studies have depicted the immune response significantly improving post-HECDI therapy, particularly CD4 + T and B cell immunity 18, 21, 27.

Previous studies have demonstrated that penicillin is an important resource for prolongation of patient's survival due to its antibacterial effect, in this case, penicillin as a hapten in chemical de-bulking of cancer prolong their life because it enhances the immunogenicity of the antigen in advanced pancreatic cancer 20, 21. That said, scientists do not understand how a strong abscopal effect, initiated by the cytotoxic drugs intratumoral injection plus penicillin (HECDI), manipulates cytokines of T-cells and/or autologous antibodies of TAAs thorough B cells to counteract metastasis 26.

## Materials and Methods

### Study Dates & Design

Participants were sorted into analogous groups by age, sex, and stages of disease into three groups. Treatments were then randomly assigned. The trial was open-label, and the control group received a placebo treatment. The study was performed at multiple sites, including Taian Cancer Hospital, Beijing Baofa Tumor Hospital, and TaiMei Baofa Cancer Hospital, from January 2016 to January 2018. Each site functioned under the same protocols, mitigating the sites' variance.

### Study Population

#### Inclusion/Exclusion Criteria

Inclusion and exclusion parameters were established for this study. Subjects of pancreatic cancer were excluded if they were less than 18 years of age. Additionally, participants were excluded if they displayed any of the following contraindications for study treatment: exhibited a poor karnofsky performance status (KPS) (≤40%), presented with a high serum total [bilirubin level > 3 mg/dL (51.3 μmol/L)] ), had any nutritional disorder(s), or suffered from renal failure [serum creatinine level > 2 mg/dl (176.8μmol/L)]. Participants were included in the study only if they received a diagnosis of pancreatic cancer with pathological diagnosis and inoperable condition. The treatment requisites for each participant was having at least one solid pancreatic tumor with a minimum diameter of 1.5 cm. The tumors were detected through CT-guided pathological evaluations performed by the study staff, before and after treatment the routine clinical tests was required.

### Treatment Groups

Table 1 illustrates the division of included participants (33) into three groups: the CDI group, the HECDI group, and the control group. Furthermore, penicillin is described as being the hapten used with the intratumoral injections for the HECDI group's therapy. At the same time, penicillin is marked as being omitted from the CDI group's therapy. Placebos are designated in Table 1 as the injection given to the control Group.

### Medical History

Table 1 denotes the demographics and disease history obtained from each subject at the start of the study. This data includes the participant's demographics and medical history. It is essential for marking progress from the before of the study wherein no CDIs, HECDIs, or placebos were administered compared with later results post treatments of CDIs and HECDIs. The participants' KPS indicates their predicted length of survival. The higher their score, the more favorable their predicted outcome. Scores are assessed based on participants' current level of functioning (capacity to support and care for oneself). The subjects' ages ranged from 50 to 80 and their KPSs ranged from 40 to 80. The presence of diabetes (Y/N), smoking status (Y/N), alcohol consumption status (Y/N), and whether the participants had prior chemotherapy (Y/N) or adjunctive therapy (Y/N). If they answered yes, they were included in the sum within their group for that factor, and if the participants responded no, they were excluded from that sum. A pathological diagnostic test was performed before any treatments were dispensed, wherein participants were noted if they were affected by pancreatic adenocarcinoma and the stage of the disease (I-IV) was specified if present. The diagnostic process performed also indicates the status of metastasized or locally advanced cancer at the start of the study and each subject's tumor size, notated as greater than 5 cm, less than 4 cm, or 4 to 5 cm.

### Preparation of the agents

One inflation device (30 atm/bar) per treatment and over 100 25-gauge spinal needles were used. The HECDI and CDI solutions were prepared at each clinical location before each injection using a uniform protocol across all sites. Each dose injected was 1 ml of drug per 1 CM3 (µg/cm3). The injection volume (ml) was calculated based on the diameter of the tumor (Dt) being treated. The Dt was multiplied by 2 for tumors ranging from 1-5 cm, and Dt was multiplied by 1.5 for tumors of 6 cm or larger. The concentrations in CDIs were 1.00 mg/ml Adriamycin (Adr), 0.80 mg/ml of cytarabine (Ara-C), and 20.0 mg/ml of H2O2. The HECDI concentrations had the were the same, but with an addition of 144 mg/ml of penicillin as hapten. Both the CDIs and HECDIs were saturated in concentration. 10 ml was administered for each dose of the CDIs and HECDIs. Doses were adjusted according to tumor size as opposed to participants' body weight.

### Treatment Site, Route, and Frequency of Administration

The skin was cleaned, and local anesthetic was applied to the area where the injection would have a short pathway to the tumor of the pancreatic organ. The spinal needle was inserted into the tumor under the guidance of CT scanning. Then the core was removed from the needle (connected to the inflator used as a high-pressure syringe), and the injection was performed. The CT allowed for confirmation of the pharmaceutical reaching the tumor. Ultrasound or CT guidance was performed to scan and monitor the density changes at the points or areas of interest in pancreatic tumors. Once every 7 days, the injection was administered as a single course, after four weeks patients need to be rechecked to decide whether or not to give an injection of therapy, or just give a single injection of CDI or HECDI, leading to the completion of therapy per participant for 60 days.

### Assessment of Treatment Efficacy

Follow-up of patients, CT scanning and blood collection for test was conducted to evaluate pancreatic treatment. Each participant's data was collected, spanning from the time of their first treatment to the instance of their death. The guidelines issued by the World Health Organization (WHO), European Organization for Research and Treatment of Cancer (EROTC), and the Response Evaluation Criteria in Solid Tumors (RECIST) were used as parameters for discernment of tumors' responsiveness to therapy before and after treatments 29-32. Thus, the disease in each participant was observed to have had a clinical response (CR), partial response (PR) or was noted as either a stable disease (SD) or progressive disease (PD) 29-32. The attending physicians filled out all case report forms (CRF). In every hospital, all physicians were trained for standard procedures. The survival statistics' relationship to the three treatments was explored, and the resulting data were analyzed for correlations.

### Antibody and Cytokine Detection Analysis

An enzyme-linked immunosorbent assay (ELISA) was used to dilute 14 purified recombinant proteins in phosphate-buffered saline (PBS). The final concentrations ranged from 0.125ug/ml to 1.0ug/ml. The proteins were then coated in a 96-well microliter plate (100ul/well) overnight at 4°C and incubated in a 1:200 diluted serum in antigen-coated wells (100ul/well) for 90 minutes at room temperature (RT). The proteins were then incubated in a 1:3000 dilution of horseradish peroxidase-conjugated goat anti-human IgG. A 2,2'-azidobis-3-ethylbenzothiazoline-6-sulfonic acid (ABTS) substrate. Then 100ul of hydrogen peroxide was added to each well. The plates were incubated without light for 10 to 15 minutes at RT. Each well's optical density (OD) value was immediately read at 405 nm on the Varioskan LUX Multimode Microplate Reader to reduce the plates' variation 33. Subsequently, 2 blank controls of 1% BSA in PBST and 8 frozen human serum samples were administered to each well of all 96-well plates. This step allowed for the normalization of different plates' OD values and adjustment of the background of all plates used 34-35. There were 507 cytokines in total, detected in the serum of the pancreatic cancer participants and healthy controls.

### Statistics

Measurements were taken seven days before each infusion and seven days after each injection (Table 2.2). The clinical benefit rate (CBR) sums the averages of participants' CR rates, PR rates, and the SD rates of those with a SD status for at least 6-months (Table 2.3) 36. The difference between the HECDI and CDI therapy arm in the frequencies of tumor responses was tested using Pearson's Chi-squared test wherein a *p*-value of < 0.05 was considered statistically significant (Table 2.3). Adverse reaction observation items and basis (Table 2.4), only a few fever and pain were observed 24 to 27% of total patients, there are a few of moderate to severe adverse reactions for all of patients (Table 2.4).

**Figure 1:**
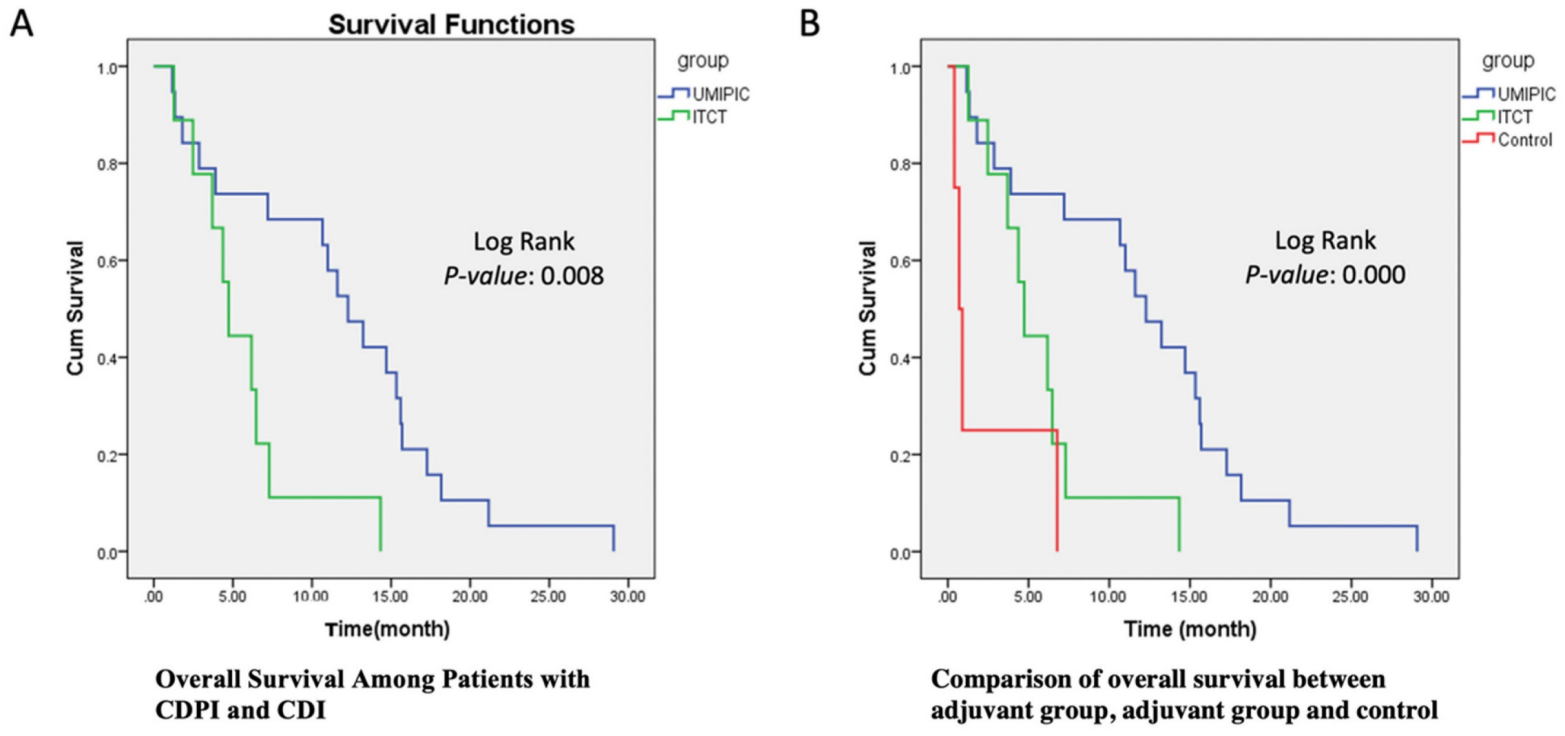
** Survival Functions.** (A) This Kaplan-Meier Plot was used to determine numerous survival data for the UMPIC (HECDI) and CDI (ITCT) Groups. The determination of the OS rate for the CDI and HECDI groups. Patient survival time in CDIP group is significant longer than CDI group. (B) Patient survival time in CDIP group is significant longer than CDI group.

The primary endpoint of overall survival (OS) was defined as the duration from the date of the first study treatment (not the date of diagnosis) to the date of death. Any participants still alive at the time of evaluation were censored. Overall survival was estimated with the use of the Kaplan-Meier methods (Figure 1A). Between-arm differences in overall survival were assessed with the help of the log-rank test (Figure 1B).

The level changes (before and after treatment) of designated cytokines and autologous antibodies of TAAs were compared in pairs of arms using Wilcoxon rank-sum (Table 3.1-3.4 & 4.1-4.4). Fold changes greater than or equal to 1.5 or less than or similar to 1 to 1.5 were considered significant. The receiver operating characteristic curve was applied to evaluate the model's performance. A leave-one-out cross-validation (LOOCV) method was used to estimate the prediction error, and the resultant calculations were then analyzed 36, 37. Statistical software SPSS version 28.0 was used for all statistical analyses 38.

The average change in IL-6, IL-10, IFN-γ, CCL3, IL-4, and IL-17A are compared in the Non-hapten (CDI), Hapten (HECDI), and Control Group for the participants who did not receive chemotherapy before study treatments (Table 3.2). An SD is listed for each value and a *p*-value < 0.05 indicates a statistically significant relationship. The participants who received chemotherapy before the study started are compared within their Non-hapten (CDI), Hapten (HECDI), and Control Groups to determine the average changes in the cytokine levels of IFN-γ**,** CCL3**,** IL-13**,** Collagen IVα1, and TIMP-1 **(**Table 3.3 & Table 3.4). SDs are listed for each level measured, and *p*-values < 0.05 signals a statistically significant difference. The participants who did and did not receive chemotherapy have their CXCL8, IFN-γ, Adiponectin, IL-13, Resistin, and Collagen Ivα1 average cytokine levels compared, with SDs listed for each group (Table 3.4). A change with a *p*-value < 0.05 is considered statistically significant.

The hapten (HECDI), non-Hapten (CDI), and control (Placebo) groups are compared against one another to assess the TAAs' autologous antibody changes after participants received all study treatments in Table 4.1. In Table 4.2 the participants who did not receive chemotherapy were each compared to one another within their hapten (HECDI), non-Hapten (CDI), and control (Placebo) Groups. Table 4.3 illustrates comparisons of participants who did receive chemotherapy within the hapten (HECDI), non-Hapten (CDI), and control (Placebo) groups. Participants were divided into those who did and did not receive chemotherapy to assess their relative changes in antibody levels in Table 4.4.

Table 5.1 lists all antibodies levels of one participant, including IMP1, Koc, p62, RalA, Survivin, Zeta, NPM1, Cmyc, p53, HCC1, and p16, which were recorded before and after the participant's first study injection. The levels are each compared with a control group participant's set of 'normal' sera values (Table 5.1). The TAA levels of a participant are recorded and compared with a bunch of 'normal' sera values from a control croup participant (Table 5.2).

## Results

### Clinical Benefits

The overall response rate (CR+PR) in the HECDI group and CDI group in table 2.2 was 30.0% and 44.4%, respectively (95% CI). The benefit rate neighbors the response rate, wherein the HECDI group's rate is 95.0%, and the CDI group's rate is 88.9% (95% CI) (Tables 2.1- 2.4). There is only a few adverse reaction and moderate severe adverse reactions happened for all of patients (Table 2.4).

### Overall survival

In Table 2.3, the overall survival probability (OS) increased by 16% in the HECDI group compared to the CDI group. The median survival time in the CDI and HECDI groups was 11.81 months and 5.64 months, respectively. The data in Table 2.3 describe a median survival time of 11.81 months for the HECDI group and 5.64 months for the CDI group. When comparing the likelihood of survival from the time of first treatment to the 6-month point, measures of dispersion indicated that HECDI treated patients were 2.27 times more likely to live longer than CDI treated participants, with 73.7% of HECDI participants and 44.4% of CDI participants surviving 6 months (Figure 1 and Table 2.3). In Table 2.3, the 6-month data were not proven to be statistically significant. Nevertheless, year-long point-in-time data analysis demonstrated a four-fold increase in survival in the HECDI group compared to the CDI group. In addition, the table conveys a comparison of survival rates in the HECDI group versus the CDI group at one year after the initial injection. The calculations showed that 52.6% of patients in the HECDI group survived during this time, compared to 11.1% in the CDI group (*p*=0.035).

**Figure 2:**
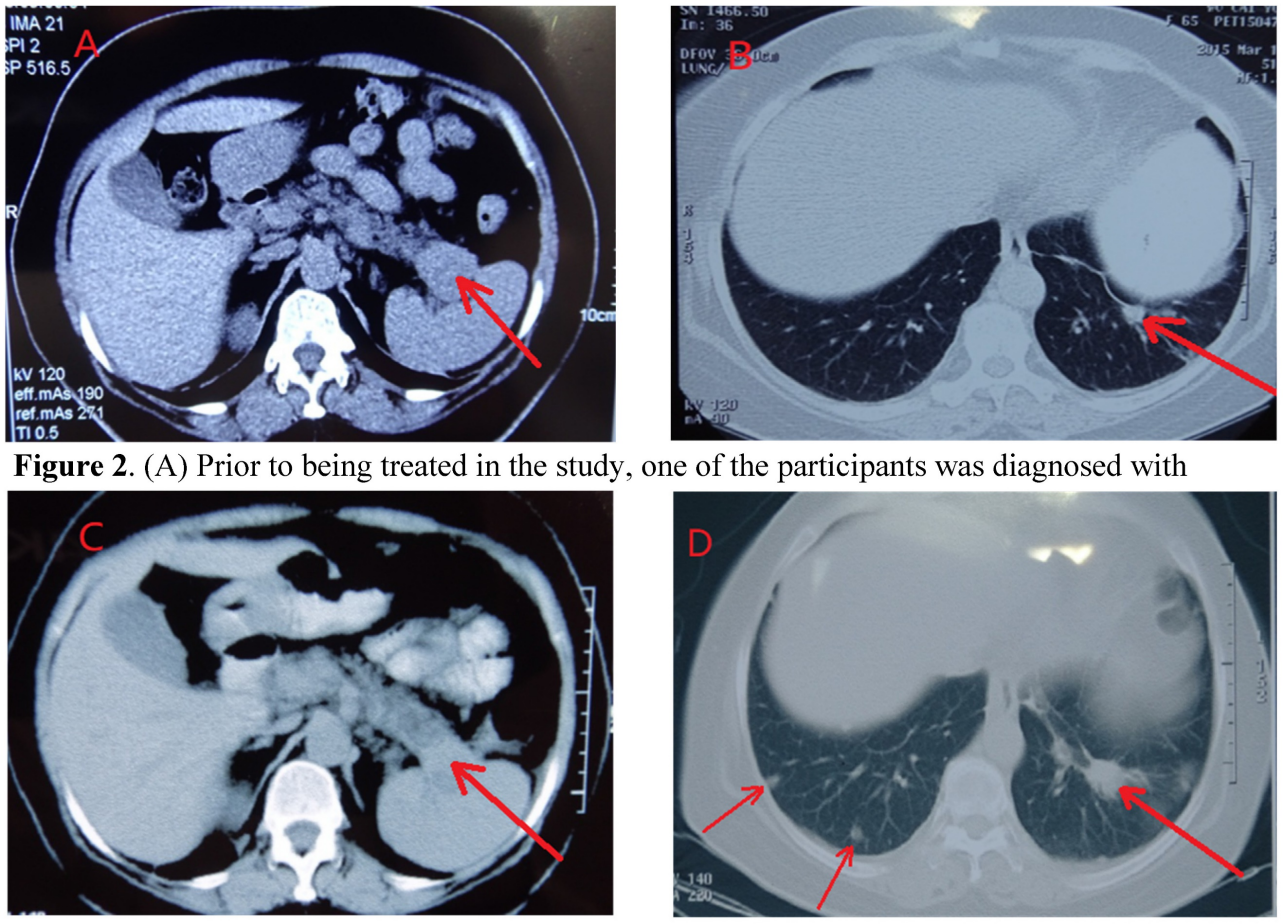
** CT of Control of Metastasis in the Lung through Systemic Immunogenicity of TAA**. (A) Prior to being treated in the study, one of the participants was diagnosed with pancreatic cancer, originating from the tail of the pancreatic organ. The tumor is at the backside of the pancreas (marked by arrow). (B) Pre-study treatment, the participant's left lung metastasized. The arrow signifies the small mass (marked by arrow) on the scan qualifies the clinical diagnosis of metastasis, without a CT or pathological examination. (C) Two months after HECDI treatment, the pancreatic tumor contracted and was subsequently stable for 2 years. (D) After two years, metastasis originating from the left lung was recognized because the mass on the participant's left lung, initially developed two years ago, presented as more substantial than the newer mass seen on the participant's right lung (right arrow). The arrows on the left indicated new metastasis in left that was not present two years ago.

The CT scans of participants throughout the course of treatment and every visit, were kept for comparisons of the location, size, and metastasis of tumors, showed a participant having a dramatic control of metastasis for up to 2 years (Figures 2A-2D). Metastasis is observable in this participant's scans, wherein their initial tumor-marked pro treatments are able to be seen two years later with unchanged growth (Figures 2A & 2C).

### Analysis of cytokines in patients with pancreatic cancer

The average change in the level of the IFN- γ, IL-12, and IL-4 cytokines among the non-hapten (CDI), hapten (HECDI), and control groups are described by their standard deviations (SDs) and compared against one another (Tables 2.1-2.4). p<0.05 indicates a statistically significant difference in the compared cytokines levels. The column to the right encompasses the HECDI versus CDI Groups' cytokines level changes (Tables 2.1-2.4). The comparison of the CDI and Control Groups, IFN-γ (94.89±42.5), IL-12 (219.94±32.35), and IL-4 (273.47±76.67) exhibit a significant level (*p*=0.034, 0.042, 0.043) (Table 3.1). A substantial increase in the level of IL-4 (168.12±51.5) in the CDI versus HECDI group is also shown (P=0.00) (Table 3.1).

Table 3.2 compares the cytokines level changes before and after CDI therapy, HECDI therapy, or placebo for participants who did not receive chemotherapy before the study. Here, among the HECDI group and control group participants, a significant increase is seen in IL-6 (10.95±3.8), IL-10 (10.3±2.61), IFN-γ (114.82±48.05), IL-4 (273.8±69.53), and IL-17A (28.56±7.36) (*p* = 0.016, 0.028, 0.012, 0.043, & 0.032). Additionally, between the CDI and the control group changes in cytokines levels preceding and following treatment in Table 3.2, a significant change in CCL3 (1509.48±371.88) is noted (*p* = 0.027). A corresponding comparison between the CDI and control groups' ratio of CCL3 (1334.11±547.68) level was also describe in Table 3.2 as undergoing a significant rise *p* = 0.044). Furthermore, the IL-6 (5.6±0.63) measure is shown to increase in the HECDI and CDI group cytokines level pre- and post-treatment comparison (*p* = 0.027)*.*

All cytokines examined in table 3.3, including IFN-γ (76.29±6.74), CCL3 (1856.82±190.66), IL-13 (16677.32±4080.45), Collagen IVα1 (2135.63±893.25), and TIMP-1 (117774.75±7485.08) revealed statically significant increase among participants in the CDI group and control group who received chemotherapy before study treatment (*p* = 0.02, 0.03, 0.04, 0.04, & 0.00). The breakdown of the HECDI group's cytokines levels versus those of the control group are detected as having a significant rise in IFN-γ (70.53±13.82), CCL3 (1824.22±521.7), and TIMP-1 (108680.37±12295.43) in table 3.3 (*p* = 0.014, 0.006, & 0.000).

By comparing the HECDI group with and without prior chemotherapy in table 3.4, the results showed that the levels of CXCL8 and IFN-γ were increased in the previous chemotherapy Group (*p* = 0.012, 0.013), while the levels of adiponectin, IL-13, Resistin, Collagen IVα1, and TIMP-1 increased in the who did not receive chemotherapy (*p* = 0.039, 0.013, 0.006, & 0.029).

### Autologous antibodies of TAAs

The comparisons of the HECDI, CDI, and control groups, no differences in the levels of IMP1, Koc, p62, RalA, Survivin, Zeta, NPM1, Cmyc, p53, HCC, and p16 are found to be statistically significant p < 0.05) (Tables 4.1-4.4). Table 4.2 describes a substantial increase in the Zeta autoantibody when the HECDI group's results versus those of the control group among those who did not receive chemotherapy before treatment p = 0.037). Table 4.3 depicts a significant rise in the IMP1 autoantibody of CDI versus HECDI therapy in those who received prior chemotherapy (*p* = 0.03). In Table 4.4, a considerable increase of the Rala, Zeta, and p16 autoantibodies is apparent following examination of the HECDI therapy participants who did not receive chemotherapy (*p* < 0.05).

### Routine clinical test

The routine clinical tests were compared before and after the treatment, it showed that there is no any significantly changes for the red, white blood cells, liver function, renal impairment (Table 2.4).

## Discussion

Pancreatic cancer falls into the category of being one of the most aggressive cancers 31. Pancreatic cancer is also associated with a high concentration of multiple drug-resistance genes 39. This outcome is supported by the fact that this novel heterogeneous approach to treating pancreatic cancer involves an increased drug concentration delivered directly to tumor sites, minimizing systemic exposure and toxicity 40. HECDI therapy being used as an alternative to surgery is auspicious, given its ability to chemically de-bulk large tumor masses, which make a ready for immune therapy since large load of tumor cut off and elicits the abscopal effect 20, 21. This effect elevates systemic immune therapeutic activities like fostering T and B cell function 26. HECDI therapy has yet to be endorsed in western countries' general clinical practices.

Due to the optimistic survival advantage of HECDI compared with CDI, a notable correlation between HECDI therapy and an extended survival period emerged (Table 2.2 & Figure 1). This relationship is most arguably attributed to its capacity to induce systemic immunogenicity of cytokines and TAAs to control tumor metastasis (Figures 1A-1C) and its predisposition to cause the abscopal effect. HECDI therapy strengthens long-term immunological memory not only T cells also B cells, which raises the magnitude of anti-tumor response proficiency 20, 21. The response is propagated by releasing fundamental antigens, antigen presenting, activate T and B cells, the produce of cytokines, and autologous antibodies of TAAs 20, 21. The surge that these components undergo coincides with the presence of the abscopal effect 19-21.

Evidence of increases in autoantibodies of TAAs after HECDI led to arguments that the autoantibodies were heavily involved in control growth of pancreatic cancers, similarity as previous report by research 12, 20, 21. Many reports about autologous antibodies of TAAs related only to the epidemiology of cancer diagnoses and consequently do not report links to cancer treatment, however, serum levels are reported to be related to cancer epidemiology 41, 42.

Incidentally, the results in this study revealed that post-HECDI, cellular cytokines increase of IL-4, IL-6, IL-12, IFN-γ, IL-17A, and increase antibodies of P53, HCC1, RalA, Zeta, and p16 genes are related to survival time. At the same time, those who did not received prior chemotherapy exhibited a higher level of cytokines of CXCL8, IFN-γ, autoantibodies of TAAs: RelA and Zeta in those non prior chemotherapy than those in the prior chemotherapy group, but those who did not received prior chemotherapy exhibited a higher level of cytokines exhibited a higher level of cytokines CXCl8 and IFN-γ while a higher of IL-3, Resistin and collagen IVal in those who received prior chemotherapy. These occurrences can be ascribed to the fact that chemotherapy has the potential to change a patient's capacity to illicit a sufficient immune response compared with those non-chemotherapy-treated participants' ability to react to the same stimulus, so that less chemotherapy may give patient's capacity to illicit a sufficient immune response for the immunotherapy.

In fact, scientists know more about cytokines and T cells related with immunity, but still do not know how autoantibodies of TAAs compete with or reach cancer cells. Researchers hypothesize that the striking results must indicate that the autoantibodies of TAAs penetrate the nucleus of cancer cells. If this is the case, they presumably engage in combat against metastasis by inhibiting or destroying their gene products. Hence, an in-depth research study to further understand the details of autoantibodies of TAAs in cancer treatment is crucial to advancing non-standard cancer therapy techniques.

## Figures and Tables

**Figure 3:**
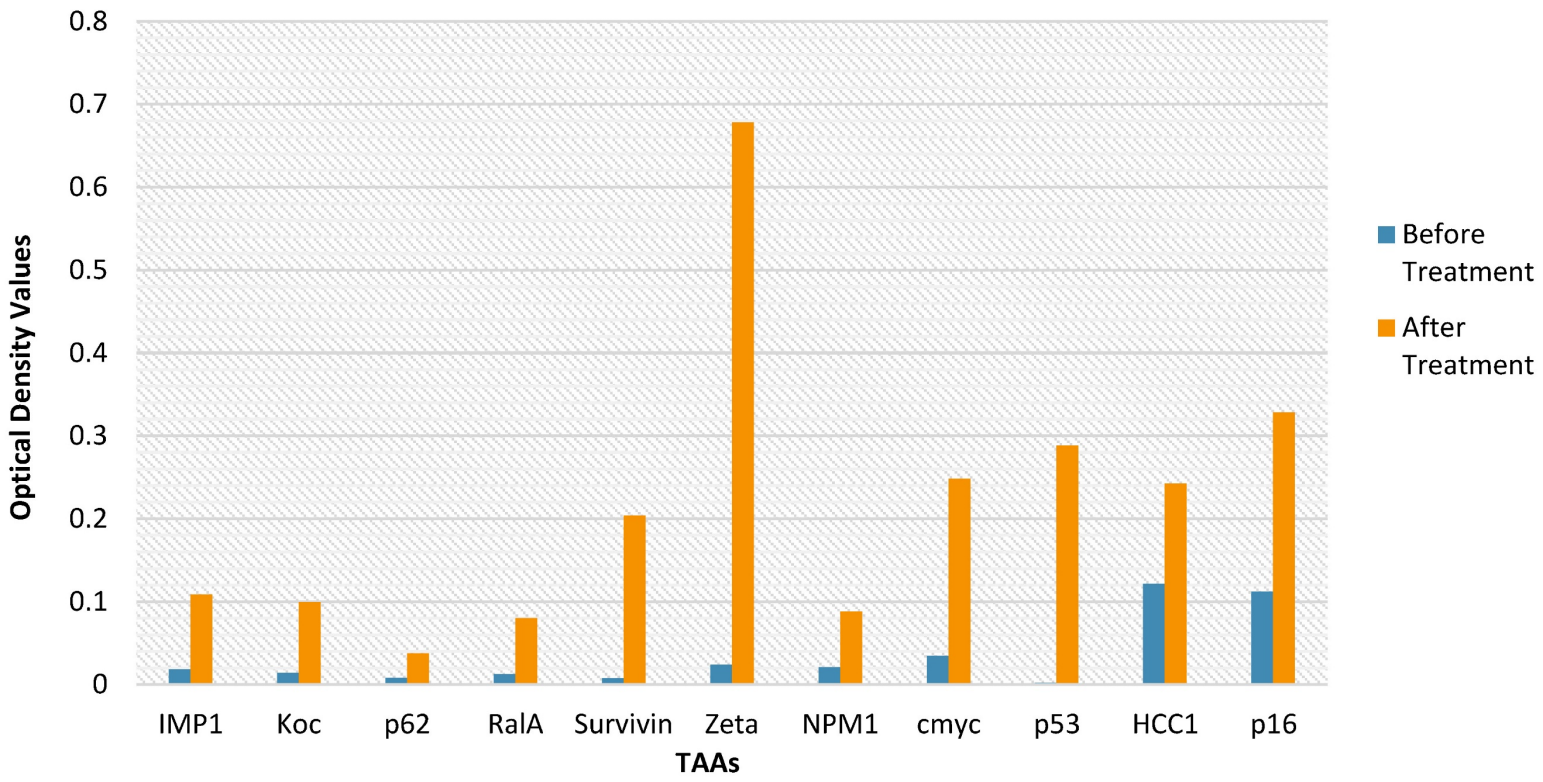
** Autologous Antibodies Before and After Therapy.** The data of a single HECDI Group participant is displayed. The level of each autologous antibody of TAA, measured from the participant's blood, is shown here before and after HECDI their 4^th^ injection.

**Figure 4:**
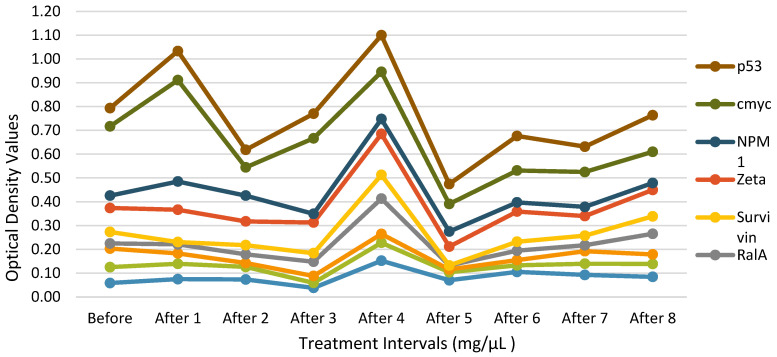
** TAA Measurements for Each Injection Time Point**. Each of the 14 purified recombinant proteins were diluted in 100 µL of phosphate-buffered saline (1:200) and incubated. Following, 100 µL of horseradish peroxidase-conjugated goat anti-human IgG was dispensed to each well on each plate dispensed (1:300) and then all the plates were incubated. The conclusive optical density (OD) values ranged from 0.0584 to 0.122.

**Table 1:** Participant Baseline Characteristics

	HECDI (N)	CDI (N)	Control group (N)
Enrolled Patients	20	9	4
Sex			
*Male*	12	3	2
*Female*	8	6	2
Age Range	50-80 (66.50±8.13)	50-70 (65.56±7.26)	50-70 (62.25±8.81)
KPS	50-80 (66.50±8.13)	50-70 (65.56±7.26)	40-70 (55.00±11.91)
Diabetes	6	2	0
Cigarette Smoking	6	3	2
Alcohol Intake	6	3	2
Stage of Disease	0	0	0
*Stage Ⅰ*	0	0	2
*Stage Ⅱ*	9	4	2
*Stage Ⅲ*	11	5	0
*Stage Ⅵ*	15	5	0
Pathology Diagnose Adenocarcinoma	15	5	0
Previous Treatment			
*Prior chemotherapy*	1	1	1
*Prior adjuvant therapy*	10	3	0
Disease status			
*Locally advanced*	10	6	0
*Metastatic disease*	6	3	0

**Table 2:** Tumor Baselines, Clinical Evaluations, and Survival Periods

**2.1 Measurements of Tumor Size by Injection Administration Times**											
Tumor Size	Before Treatment	Before Treatment	Before Treatment	Before Treatment	Before Treatment	Before Treatment					
< 4cm	6	9	1	3	3	no					
4-5 cm	6	6	4	2	0	no					
>5 Cm	7	4	4	4	1	no					
**2.2 Comparison of HECDI vs CDI Therapeutic Effect**											
Groups	N	CR	PR	SD	PD	Response Rate (%)	Benefit Rate (%)	P			
HECDI	20	0	6	13	1	30	95.00	> 0.05			
CDI	9	0	4	4	1	44.44	95.00	88.89			
**2.3 Comparison of HECDI vs CDI Survival Periods**											
Groups	N	Mean Survival /Month	Median Survival /Month	Log Rank Chi-Squared	P	6 Month Survival Rate	Chi- Squared	P	12 Month Survival Rate (%)	Chi- Squared	P
HECDI	19	11.81	12.27	0.16	> 0.05	73.68	2.27	> 0.05	52.63	4.41	= 0.035
CDI	9	5.64	4.37			44.44			11.11		
**2.4 Adverse reactions of pancreatic cancer after therapy (Notes)**											
Kind of Adverse reaction	Fever	Pain	Leukopenia	Hemoglobin reduction	Thrombocytopenia	Liver function damage	Renal impairment	Nausea	Vomiting	Rash	Hair loss
Total Cases	29	29	29	29	29	29	29	29	29	29	29
Adverse reaction (%)	8 (27.5 %)	7(24.1 %)	5(15.74 %)	1(3.4 %)	1(3.4%)	0(0%)	0(0%)	0(0%)	1(3.4%)	1(3.4%)	0(0%)
Moderate Severe Adverse Reactions (%)	2(6.89 %)	1(3.4%)	1(3.4%)	0(0%)	0(0%)	0(0%)	0(0%)	0(0%)	0(0%)	0(0%)	0(0%)

Notes: According to the US Department of Health and Public Health's Common Adverse Event Evaluation Criteria (CTCAE) (released on November 27, 2017, version 5.0), selection of white blood cells, hemoglobin, platelets, liver function, renal function, nausea, vomiting, rash, neurotoxicity.

**Table 3:** Analysis of Cytokines Before and After HECDI, CDI, and Placebo Treatments

3.1 Comparison Between the Hapten, Non-Hapten and Control Group						
Name of Cytokines	Non-Hapten (CDI) N=8	Hapten (HECDI) N=20	Control N=3	P_CDI VS Control_	P_HECDI VS Control_	P_CDI vs HECDI_
IFN-γ	83.96±9.72	94.89±42.5↑	46.04±8.67	0.125	0.034	0.467
IL-12	220.12±32.27↑	219.94±32.35↑	177.92±24.29	0.06	0.042	0.989
IL-4	196.45±95.33	273.47±76.67↑↑	168.12±51.5	0.607	0.043	0.00
**3.2 Comparison Between Participant Without Prior Chemotherapy**						
Name of Cytokines	Non Hapten (CDI) N=6	Hapten (HECDI) N=11	Control N=3	P_CDI VS Control_	P_HECDI VS Control_	P_CDI VS CDIP_
IL-6	7.18±1.67	10.95±3.8↑↑	5.6±0.63	0.474	0.016	0.027
IL-10	9.47±1.78	10.3±2.61↑	6.46±3.06	0.102	0.028	0.511
IFN-γ	86.52±9.58	114.82±48.05↑	46.04±8.67	0.144	0.012	0.154
CCL3	1509.48±371.88	1334.11±547.68	775.27±309.07	0.044	0.090	0.479
IL-4	207.02±89.72	273.8±69.53↑	168.12±51.5	0.469	0.043	0.095
IL-17A	23.07±3.16	28.56±7.36↑	21.61±0.75	0.692	0.032	0.141
**3.3 Comparison Between Participants With Prior Chemotherapy**						
Name of Cytokines	Non Hapten (CDI) N=2	Hapten (HECDI) N=9	Control N=3	P_CDI VS Control_	P _HECDI VS Control_	P_CDI VS CDIP_
IFN-γ	76.29±6.74	70.53±13.82	46.04±8.67↓	0.023	0.014	0.568
CCL3	1856.82±190.66	1824.22±521.7	775.27±309.07↓	0.028	0.006	0.931
IL-13	16677.32±4080.45	16377.38±2872.51	10971.67±564.42↓	0.044	0.013	0.892
Collagen IVα1	2135.63±893.25	1419.67±810.72	519.16±130.28↓	0.037	0.097	0.244
IMP-1	117774.75±7485.08	108680.37±12295.43	59912±6552.02↓	0.000	0.000	0.316
**3.4 Comparison Between Participants With and Without Prior Chemotherapy HECDI Group**						
Name of Cytokines	No Prior Chemotherapy N=11	Prior Chemotherapy N=9	P			
CXCL8	332.67±289.52↑	63.33±44.59	0.012			
IFN-γ	114.82±48.05↑	70.53±13.82	0.013			
Adiponectin	2355446.61±965403.62	3116080.08±372628.73	0.039			
IL-13	11384.59±4737.35	16377.38±2872.51↑	0.013			
Resistin	112550.05±96647.63	11653.5±11619.57↑	0.006			
Collagen IVα1	753.47±422.63	1419.67±810.72↑	0.029			

**Table 4:** Comparisons of Autologous Antibodies of TAAs' Levels

**4.1 Comparison of the Hapten, Non-hapten and Control Group**						
Name of Genes	Hapten (HECDI) N=20	Non-Hapten (CDI) N=9	Control N=4	P_CDI VS Control_	P_HECDI VS Control_	P_CDI VS CDIP_
IMP1	0.111±0.047	0.193±0.194	0.098±0.019	0.145	0.818	0.064
Koc	0.074±0.060	0.182±0.326	0.058±0.023	0.641	0.752	0.731
p62	0.029±0.025	0.025±0.019	0.032±0.028	0.589	0.820	0.618
RalA	0.058±0.039	0.047±0.028	0.059±0.023	0.576	0.975	0.429
Survivin	0.057±0.067	0.073±0.057	0.051±0.026	0.563	0.858	0.533
Zeta	0.080±0.054	0.203±0.239	0.050±0.027	0.254	0.343	0.416
NPM1	0.072±0.038	0.103±0.089	0.046±0.027	0.095	0.393	0.172
Cmyc	0.285±0.736	0.173±0.129	0.165±0.093	0.981	0.713	0.641
p53	0.251±0.733	0.164±0.158	0.133±0.131	0.931	0.717	0.716
HCC1	0.128±0.056	0.168±0.067	0.180±0.036	0.733	0.113	0.098
p16	0.108±0.080	0.171±0.131	0.124±0.057	0.418	0.768	0.112
**4.2 Comparison of Participants without Prior chemotherapy Before and After of HECDI and CDI Therapy**						
Name of Genes	Hapten (HECDI) N=11	Non-Hapten (CDI) N=7	Control N=4	P_CDI VS Control_	P_HECDI VS Control_	P_CDI VS CDIP_
Zeta	0.108±0.043	0.253±0.250↑	0.050±0.027	0.503	0.037	0.05
**4.3 Comparison of Patients with Prior Chemotherapy Before and After HECDI and CDI Therapy**						
Name of Genes	Hapten (HECDI) N=9	Non-Hapten (CDI) N=2	Control N=4	P_CDI VS Control_	P_HECDI VS Control_	P_CDI VS CDIP_
TIMP1	0.107±0.056	0.197±0.004↑	0.098±0.019	0.031	0.748	0.031
NPM1	0.055±0.017	0.085±0.002	0.046±0.027	0.037	0.425	0.074
HCC1	0.103±0.052	0.071±0.009	0.180±0.036	0.018	0.042	0.322
**4.4 Comparison of Patients with and Without Prior Chemotherapy After HECDI Therapy**						
Name of Genes	Prior Chemotherapy N=9	Non-Prior Chemotherapy N=11	P			
RalA	0.039±0.035	0.074±0.035↑	0.038			
Zeta	0.047±0.047	0.108±0.043↑	0.007			

**Table 5:** Antibodies and TAA Levels

**5.1 Antibodies (μg/mL) Before injection and After First Injection**														
Date	Treatment	Sera ID	IMP1	Koc	p62	RalA	Survivin	Zeta	NPM1		Cmyc	p53	HCC1	p16
09.14.2016	0	25	0.0584	0.0668	0.0778	0.0215	0.0485	0.1005	0.052		0.2913	0.0766	0.1659	0.0727
09.9.2016	1	26	0.0746	0.0645	0.0437	0.0381	0.0094	0.1361	0.1185		0.426	0.1221	0.1469	0.1773
09.10.2016	1	27	0.0732	0.0529	0.0163	0.0366	0.0383	0.0999	0.1083		0.1187	0.0737	0.1589	0.1265
09.10. 2016	1	28	0.038	0.0209	0.0292	0.0596	0.0361	0.1287	0.0366		0.3173	0.1035	0.0696	0.1205
09.10. 2016	1	29	0.1522	0.0753	0.0373	0.1482	0.0994	0.1725	0.0628		0.1976	0.1543	0.1103	0.227
09.12. 2016	1	30	0.0699	0.0336	0.0118	0.0169	0	0.0791	0.0636		0.116	0.0836	0.0953	0.0733
09.4. 2017	1	31	0.105	0.0275	0.0223	0.0395	0.0376	0.127	0.0382		0.134	0.145	0.1847	0.0929
09.10. 2017	1	32	0.0924	0.0469	0.0529	0.0249	0.0401	0.0825	0.0384		0.1468	0.1065	0.1211	0.1236
09.10.2017	1	33	0.0842	0.0544	0.04	0.0865	0.0733	0.1112	0.0289		0.1313	0.1538	0.0699	0.1267
**5.2 TAAs' Levels (μg/mL) at first and last Treatment Time Interval**														
Date	Treatment	Sera ID	IMP1	Koc	p62	RalA								
4.19.2016	0	15	0.0185	0.0139	0.0081	0.0122								
6.1.2016	1	16	0.1085	0.0995	0.0374	0.0801								
														

## Abbreviations

CDI: cytotoxic drugs intratumoral injection
HECDI: hapten-enhanced cytotoxic drugs intratumoral injection
CCRT: concurrent chemo or radiotherapy
WtoS: week to sick
